# The Operative Treatment of Roaring

**Published:** 1890-09

**Authors:** 


### THE OPERATIVE TREATMENT OF ROARING.[1]

If there had been any lingering hopes that the revived operative treatment of roaring would prove beneficial, notwithstanding the unfavorable results published by a few operators, these hopes would have been dispelled by the information made public in Mr. Dollar's paper. Mr. Dollar's paper is not pleasant reading. It is impossible not to regret that the operation, regarding which many had been led to form sanguine expectations, chiefly in consequence of the very favorable results published by Dr. Möller, of Berlin, has, after extended trial on the army horses, proved to be worse than a failure. But there is still greater reason to regret the manner in which the details regarding these army operations have been brought to light.

It will be recollected that, during the early part of 1888, the veterinary profession learned, through the columns of the lay press, that the principal veterinary surgeon to the British Army had devised a remedy for roaring. The vehicles selected to make public the few meagre details vouchsafed regarding the nature of this remedy were, for the most part, daily newspapers and agricultural journals, and it almost appeared as if an attempt was being made to whet public curiosity by enveloping the affair with an air of mystery. It soon leaked out, however, that the reported new discovery consisted in excision of the left vocal cord. It was subsequently pointed out to Dr. Fleming, in these columns[2] and elsewhere, that this was not a new operation, and, furthermore, that there was small reason to hope that it would accomplish the object in view, since the paralyzed vocal cord was certainly not the only, nor even the main, cause of the abnormal respiratory sound. Following close upon that came the publication of Dr. Möller's monograph on roaring, and the results therein stated to have been obtained by resection of the left arytenoid cartilage, combined

[1] Editorial from Journal of Comparative Pathology and Therapeutics, June, 1890.
[2] *Ibid.*

with the use of a tampon tracheotomy tube during the healing of the wound, certainly appeared to justify the hope that roaring would be brought within the sphere of curable diseases.

Dr. Möller's method of operating was now adopted in the treatment of the army horses, and the result of the operations was awaited with considerable interest. Strange to say, no precise details regarding these army operations were forthcoming. Nor could this reticence be set down to a commendable unwillingness to anticipate the result of an operation in which permanent, as opposed to immediate and possibly transitory, improvement must form the criterion of success, for the profession was given to understand that the army operations were a decided triumph, and marked an epoch in the history of veterinary surgery.

Dr. Fleming's attitude in connection with these operations was subjected to a good deal of hostile criticism, and Mr. J. A. W. Dollar was certainly the author of some of the most caustic comments which, on the other hand, called forth some very forcible, though rather undignified, rejoinders by Dr. Fleming. The result of this literary encounter was that Dr. Fleming, in the month of March of last year, brought an action for libel against Mr. Dollar, the damages claimed being £5000. In defence, the latter asserted that the statements complained of were fair comment on a matter of public interest, and true in substance and in fact, and he brought a counter-claim for £500 in respect of certain statements made regarding him by Dr. Fleming in the correspondence above referred to.

Into the merits of these actions we have no desire to enter, and we refer to them here merely in order to show the manner in which the information conveyed in the table annexed to Mr. Dollar's paper was brought to light. The actions, it may be noted, were abandoned on Dr. Fleming agreeing to pay Mr. Dollar £300 in satisfaction of costs and expenses incurred. In the action, at the instance of Dr. Fleming, the latter was directed, by an order of the court, to furnish answers to certain interrogatories delivered on behalf of the defendant. These answers are given in the column headed "Results as Stated by Dr. Fleming on Oath." In the same action, at the instigation of the defendant, there were submitted to inspection the official sheets at the War Office relating to the operations for roaring practiced on the army horses. The column headed

"Results as shown by Official Returns" is compiled from this source.

The discrepancy between these two columns is calculated to strike one with astonishment. Take, for example, the case of the horse marked No. 20 in the table. In his sworn answers to interrogatories, made on the 29th of November last, Dr. Fleming stated: "I first superintended an operation on two horses affected with roaring, at Aldershot, on the 19th of January, 1888, when Veterinary Surgeon F. Smith, of Aldershot, was the operator. The operations performed on the two horses, which were both army horses and badly affected, varied. In one case the left vocal cord was extirpated by an opening made through the thyroid ligament between the wings of the thyroid cartilage, and the arytenoid cartilage was subsequently removed. This horse was completely cured by the operation described. This horse so cured was No. 26, Troop A, Royal Dragoons. It was sold by auction on August 31st, 1889, on account of constitutional weakness. I do not know, and have no means of knowing, where it is now." On turning to the official record regarding this horse at the War Office, the following statement is found: "Operation for Roaring—Incurable," to which is appended the signature of Veterinary Surgeon Shortus.

A similar grave discrepancy will be noticed in the case of Nos. 10, 20, 21, 22, 24, 25, 26, 37, 38, 44 and 66, and in several of these cases the official record states explicitly that the horse was sold on account of roaring. Furthermore, in a number of instances Dr. Fleming omits to state the result, or gives it in such terms as "sold by auction," while the official sheets record the horse as having proved incurable.

We say that these discrepancies are calculated to strike one with astonishment. It would be vain to attempt to explain them. A simple explanation would be that Dr. Fleming, not knowing that the official records of the operation were to be submitted to inspection, deliberately falsified the results, but everyone will agree with us that that supposition is almost incredible, and certainly not to be entertained so long as any other explanation is possible. It is quite as difficult to believe that Dr. Fleming's subordinates were guilty of either ignorance or untruthfulness in filling in the record regarding the horses operated upon, or that Dr. Fleming, under oath, asserted that such and such were the results of these operations, without having taken the trouble to ascertain the condition of the horses at the moment when they were sold or destroyed. Meanwhile, the veterinary profession will await Dr. Fleming's explanation; for explain he must, unless he wishes the worst possible construction to be put upon the matter.

